# Soil Porosity Detection Method Based on Ultrasound and Multi-Scale Feature Extraction

**DOI:** 10.3390/s25103223

**Published:** 2025-05-20

**Authors:** Hang Xing, Zeyang Zhong, Wenhao Zhang, Yu Jiang, Xinyu Jiang, Xiuli Yang, Weizi Cai, Shuanglong Wu, Long Qi

**Affiliations:** 1 College of Engineering, South China Agricultural University, Guangzhou 510642, China; xinghang@scau.edu.cn (H.X.); zeyangzhong@stu.scau.edu.cn (Z.Z.); wenhaozhang@stu.scau.edu.cn (W.Z.); xinyu_jiang@stu.scau.edu.cn (X.J.); xlyscau@scau.edu.cn (X.Y.); wzcoi@scau.edu.cn (W.C.); 2 Information Network Center, South China Agricultural University, Guangzhou 510642, China; nova_yy@scau.edu.cn; 3 College of Water Conservancy and Civil Engineering, South China Agricultural University, Guangzhou 510642, China; 4 State Key Laboratory of Agricultural Equipment Technology, Guangzhou 510642, China; 5 Department of Biosystems Engineering, University of Manitoba, Winnipeg, MB R3T 5V6, Canada; 6 Guangdong Engineering Technology Research Center of Rice Transplanting Mechanical Equipment, Guangzhou 510642, China

**Keywords:** soil porosity, ultrasonic testing, multi-scale feature extraction, large convolution kernel

## Abstract

Soil porosity, as an essential indicator for assessing soil quality, plays a key role in guiding agricultural production, so it is beneficial to detect soil porosity. However, the currently available methods do not apply to high-precision and rapid detection of soil with a black-box nature in the field, so this paper proposes a soil porosity detection method based on ultrasound and multi-scale CNN-LSTM. Firstly, a series of ring cutter soil samples with different porosities were prepared manually to simulate soil collected in the field using a ring cutter, followed by ultrasonic signal acquisition of the soil samples. The acquired signals were subjected to three kinds of data augmentation processes to enrich the dataset: adding Gaussian white noise, time shift transformation, and random perturbation. Since the collected ultrasonic signals belong to long-time series data and there are different frequency and sequence features, this study constructs a multi-scale CNN-LSTM deep neural network model using large convolution kernels based on the idea of multi-scale feature extraction, which uses multiple large convolution kernels of different sizes to downsize the collected ultra-long time series data and extract local features in the sequences, and combining the ability of LSTM to capture global and long-term dependent features enhances the feature expression ability of the model. The multi-head self-attention mechanism is added at the end of the model to infer the before-and-after relationship of the sequence data to improve the degradation of the model performance caused by waveform distortion. Finally, the model was trained, validated, and tested using ultrasonic signal data collected from soil samples to demonstrate the accuracy of the detection method. The model has a coefficient of determination of 0.9990 for detecting soil porosity, with a percentage root mean square error of only 0.66%. It outperforms other advanced comparative models, making it very promising for application.

## 1. Introduction

As a substance in large quantities on the earth, soil has many uses and plays a vital role in providing fuel, water resources, building materials, and crops [1]. Therefore, soil quality is related to all aspects of economic efficiency and industrial production, especially in agriculture. Maintaining soil quality is a priority of agricultural policy in the European Union [2], and the conservation and circulation of water, the abundance of organisms in the soil, soil porosity, and the stability of soil aggregates [2,3,4,5] are the main factors affecting soil quality. As an essential part of soil quality assessment, soil porosity affects many aspects such as soil hydraulic conductivity, water retention, air permeability, and soil looseness [2,6,7,8,9], which are all closely related to vegetation growth. Therefore, rapid detection of soil porosity is a key step in agricultural production.

There are various methods for detecting soil porosity, and the traditional testing methods are usually categorized into laboratory testing methods and field testing methods, which have advantages and disadvantages. Among the laboratory testing methods, the pressed mercury method [10] has a short measurement time and a wide measurement range, but it cannot detect closed and isolated pores. The volumetric method [11] is simple and low-cost, but it is too time-consuming and cannot achieve real-time detection. Non-destructive three-dimensional imaging methods, such as computed tomography [12], can obtain three-dimensional structural information of soil pores in a non-destructive way. Still, the equipment is expensive, and the operation is complicated. The thermal pulse time-domain method [13] among the field testing methods can monitor the changes in soil porosity in real time on site but is prone to errors due to the oscillation of the probe into the soil. The black-box nature of the soil causes most soil porosity detection methods to have defects, such as cumbersome detection steps, low efficiency, and damage to the soil structure, so there is an urgent need for a fast and low-cost detection method with penetrability. Ultrasonic waves have very strong penetrability and can penetrate solids, liquids, and gases easily. At present, the ultrasonic method is widely used in the detection of composite or metal materials and is one of the most widely used nondestructive testing methods. With the development of computer technology, traditional machine learning methods are widely used in ultrasonic nondestructive testing to improve the accuracy and automation level of defect recognition. Compared with the analysis method that completely relies on manual experience, machine learning can assist in determining whether there are defects inside the material based on effective features extracted from a large number of ultrasonic signals. This combination not only improves the accuracy of defect recognition but also significantly enhances the automation and intelligence level of the inspection process. Francirley Paz da Silva [14] et al. used three traditional machine learning methods to detect carburization damage in three different regions of the furnace tube using ultrasonic signals. Ultrasonic signals in A-scan format were obtained for all the samples at a frequency of 5 MHz. Then, their features were extracted using FFT to classify and evaluate the classifier’s performance, proving the effectiveness of the combination of ultrasonic detection and machine learning for detecting carburization in HP steel furnace tubes. Jinrui Zhang [15] et al. conducted electrochemical accelerated corrosion tests and ultrasonic detection monitoring on reinforced concrete specimens with 0, 10%, and 20% rubber content, respectively. Six machine learning models were trained using ultrasonic inspection data to predict the corrosion degree of rubberized concrete based on the ultrasonic features. The results showed that the machine learning models, except for the linear model, could predict the corrosion degree accurately and robustly under the disturbances of the outliers’ magnitude and the training set’s size. Navya Prakash [16] et al. utilized state-of-the-art machine learning algorithms, support vector machines, and decision trees for the offline quality assessment of NDT-FML of A380 aircraft structures to identify defects in ultrasonically detected scanned images. These models are embedded with different image feature extraction techniques, SURF and HoG. The combination of HoG-Linear SVM and SURF-Decision Fine Tree outperforms all the other models, suggesting that the methodology is useful to inspectors for quality control and assurance of the aircraft production process and is important for NDT 4.0. Of course, ultrasound also has a place in soil research. Ultrasound can carry a lot of information about the physical properties of soil after penetrating or being reflected by the soil. Therefore, by analyzing the received ultrasound signals, the soil’s porosity, texture, moisture, and other important physical properties can be deduced. Stuart G. Bradley [17] et al. utilized a novel non-contact ultrasonic method to estimate the porosity of agricultural soils by analyzing ultrasonic pulses reflected from the soil surface; Wang, S. [18] et al. analyzed the feasibility of amplitude as an expression of geotechnical strength damage by taking the first-wave amplitude as the damage state variable, confirming and generalizing the quantitative relationship between the mechanical properties of the geotechnical materials and the ultrasonic parameters. Meanwhile, some scholars have also attempted to utilize machine learning algorithms to assist in conducting ultrasonic testing research on soils. Umut Orhan [19] et al. proposed an automated soil texture analyzer supported by a microcomputer and a machine learning approach, applying a feature extraction step to extract the features of ultrasonic waves that penetrate the soil–water mixtures as inputs to machine learning to predict the soil’s sand, dust, and clay components in the soil. Although the accuracy does not seem to be very high, one of the main purposes of the device is to be used in situ. These results were obtained only by applying Calgon preprocessing, and the comparison is to a machine worth thousands of dollars, so the proposed device can be used as an alternative; Dong Kook Woo [20] et al. measured the leakage Rayleigh waves at the half-space of air–soil joints and found there is a strong correlation between the energy and amplitude of the leakage Rayleigh waves and soil moisture, and a random forest model is used to predict soil moisture.. Table 1 summarizes the above studies using machine learning to assist ultrasound detection. Although traditional machine learning methods have achieved certain application results in ultrasonic nondestructive testing, especially in defect classification and identification tasks, which show higher accuracy and stronger generalization ability compared with manual rules, these methods still face many limitations, which restrict their further promotion and application in complex industrial scenarios. First, traditional machine learning is highly dependent on the quality of feature engineering. Its performance largely depends on the manually extracted feature set, which often requires the knowledge of domain experts and the accumulation of many experiments. It is difficult to adapt to the changes in signal properties in different materials, structures, or detection environments. Secondly, such methods are usually only applicable to small-scale datasets and show obvious performance bottlenecks when facing high-dimensional, nonlinear, and noise-interfering ultrasound data. In addition, most traditional algorithms cannot model temporal information and spatial context, making it difficult to capture deep structural features in the signal.

To overcome the above problems, artificial neural networks (ANN) have been widely used as a powerful computational model for ultrasonic detection in recent years. Its core advantage lies in its ability to learn directly from the relationship between ultrasonic signal waveforms and material properties through the training process, without the need for an in-depth understanding of the specific physical mechanisms of ultrasonic signal propagation in the material. Its good self-learning and self-adaptation capabilities enable it to automatically adjust the model parameters according to different input data, thus theoretically reducing the impact of human factors and uncertainty on the inspection results, and better adapting to the complex and changing inspection environment. Currently, defect characterization based on deep learning and ultrasound is widely used in various fields, YongMin Guo [21] et al. proposed an inspection baseline model based on full convolutional networks and gated loop units for classifying ultrasound signals of 3D woven composite samples with debonding defects; Iman Ranjbar [22] et al. compared the manual extraction of damage-sensitive features and automatic extraction of damage-sensitive features through LSTM layers for damage assessment of concrete, and the results showed that using the time series of ultrasonic response signals as inputs to the LSTM model was superior to using manual extraction of the features. Seong-Hyun Park [23] et al. utilized time-domain ultrasonic signals obtained from additively fabricated samples with different porosities and surface roughnesses for the multiple neural network models, which were trained, and the results showed that all of them were able to evaluate the porosity accurately. CNN-LSTM, as a classic model of artificial neural network, is a deep learning architecture that combines the advantages of a convolutional neural network (CNN) and a long short-term memory network (LSTM). CNN can perform feature extraction and automatically learns effective feature representations in the data, which is especially good at processing data with spatial structure. At the same time, LSTM is good at sequence data modeling and can capture temporal dependency and long-term memory information in the data. The combination of their ability to deal with time and space can achieve a more comprehensive and in-depth understanding and modeling of the input data. Therefore, CNN-LSTM models have been widely used in ultrasonic NDT and have achieved excellent results. Li Shang [24] et al. designed different damage states in the COMSOL environment and simulated local damage detection using ultrasound-guided waves to generate different case training data, which were fed into SVM and CNN-LSTM models. The results showed that the accuracy and robustness of the deep learning model of CNN-LSTM are much higher than those of the shallow learning model of SVM. Using the finite element method (FEM), Junzhen Wang [25] et al. simulated the probing of a two-dimensional double-layer plate using ultrasonic guided waves in the hope of developing a reliable ultrasonic nondestructive testing technique to characterize delaminations in double-layer plates and to non-destructively locate the position and dimensions of the delaminations at the bonding interface. In the paper, a customized CNN-BiLSTM model is used to predict the test set and compared with CNN, LSTM, BiLSTM, and CNN-LSTM, and from the prediction results, the accuracy of CNN-BiLSTM and CNN-LSTM is comparable and far better than the other three models. Weihan Shao [26] et al. proposed a multitask CNN-LSTM damage quantization method combined with transfer learning, where two parallel branches in the multitask CNN-LSTM can simultaneously output the damage coordinates in the x and y directions to locate the damage at any position within the structure. The method directly utilizes the original discrete time-domain Lamb wave signals to predict the size and location of the damage, and the pre-trained model on one plate is implemented to identify the location of structural damage on the other two plates using a transfer learning method. These studies demonstrated that artificial neural networks are effective in the task of defect detection of different materials using ultrasound, however, few studies have been conducted on ultrasonic detection of soil porosity using artificial neural networks based on artificial neural networks.

Therefore, this study proposes a method based on ultrasound and artificial neural network that can detect soil porosity quickly and accurately. We constructed and optimized an artificial neural network model based on multi-scale CNN-LSTM for the characteristics of the data obtained from the ultrasonic detection of soil. The research process is as follows: first, a series of standard soil samples with different porosities were prepared manually, and ultrasonic signals were collected from these samples. To enrich the dataset, the acquired signals were processed with data augmentation. Subsequently, a multi-scale CNN-LSTM model was constructed for the characteristics of ultrasonic signals with different frequency and sequence features, and optimized and improved based on the severe distortion of soil ultrasonic signals. After completing the training and validation of the model, the trained model was used to evaluate the test set and compared with other models, and the results proved the effectiveness and accuracy of the method in soil porosity detection.

## 2. Materials and Methods

### 2.1. Preparation of Soil Samples

**Figure 1:**
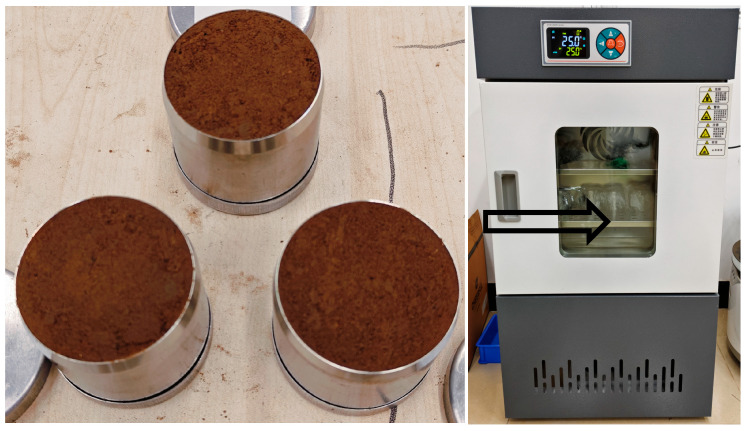
Preparation of soil samples for experiments. From left to right: Prepared standard soil samples, and insulation of soil samples.

The soil used in this experiment was a wheat tillage soil from Huimin County, Shandong Province, China, which was collected from shallow cultivated soil at a depth of 5–20 cm with a particle density of 2.43 g/cm^3^. The particle distribution of the soil was as follows: 164 g/kg for sand (2.0–0.05 mm), 608 g/kg for powder (0.05–0.002 mm), and 228 g/kg for clay (<0.002 mm), which is classified as powdery loam. We collected 5 kg of raw soil using shovels over an area of approximately 10 m^2^ for subsequent processing. The collected soil was heated in a heating chamber at 105 °C for 24 h to dry it out completely. Then the weeds and large stone particles in the soil were sieved out by using a sieve with a 2 mm aperture to exclude the influence of other factors on the ultrasonic signals, so that it is possible to focus on the coupling that exists between the soil porosity and the ultrasonic signals. Then, the soil samples were prepared using a soil porosity test based on the volumetric method to determine the specified porosity and water content. Finally, a soil porosity test based on the bulk density method was used to prepare soil samples with specified porosity and water content [27], with the following equations:

(1) $$\theta =1-\frac{p_{b}}{p_{s}},$$

(2) $$p_{b}=\frac{100\times m_{w}}{V(100+\sigma )},$$

(3) $$\sigma =\frac{m_{w}-m_{d}}{m_{d}},$$

where θ is porosity, $p_{b}$ is bulk density, $p_{s}$ is soil particle density, $m_{w}$ is the mass of wet soil with water, V is the soil volume, σ is the water content, and $m_{d}$ is the mass of dry soil. The weighed soil and water were mixed well and poured into a 100 cm^3^ ring cutter, and the soil was pressed thoroughly into the inside of the ring cutter using a hydraulic press to simulate the morphology of soil samples collected from agricultural fields. To avoid the influence of variables such as water content and ambient temperature on the experimental results, the water content was set at 18%, and the prepared soil samples were placed in an insulated box at 25 °C for 24 h, as shown in Figure 1. The ring cutters were wrapped with plastic wrap to prevent water evaporation. Thirteen soil samples with porosities of 30%, 32.5%, 35%, 37.5%, 40%, 42.5%, 45%, 47.5%, 50%, 52.5%, 55%, 57.5%, and 60% were prepared, which covered the common porosity sizes in agricultural soils to ensure the generalizability of this soil porosity testing method.

### 2.2. Ultrasonic Data Acquisition

When ultrasound waves are emitted from a source into a soil sample, the soil particles’ irregularity and the composition’s non-uniformity can lead to complex interactions of the ultrasound waves during propagation [28]. Ultrasound waves will encounter different acoustic impedances at the interfaces of varying soil particles, resulting in partial reflection and scattering of the sound waves. Some of the reflected ultrasounds will return along the original propagation path, while the other part will continue to propagate forward through different interfaces. The scattered ultrasound will continue propagating through the soil along more complex paths, where some of the energy will be converted into heat and dissipated into the air, while others may eventually be captured by the ultrasonic receiver transducer [29]. Therefore, the ultrasonic signals received through soils with different porosities will be different.

**Figure 2:**
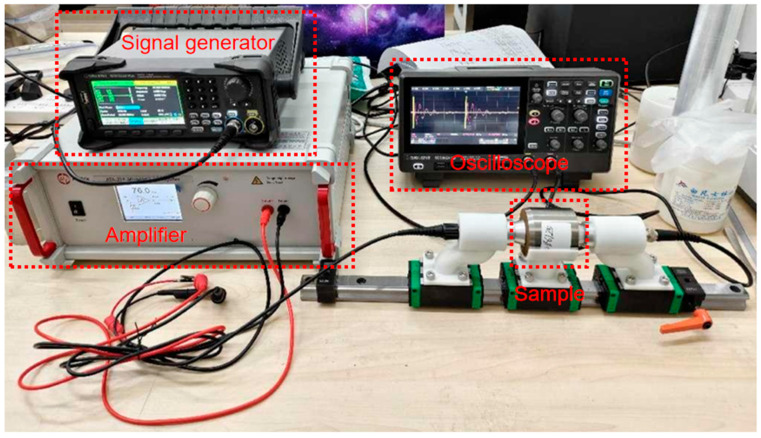
Equipment for ultrasound data acquisition.

The ultrasonic data acquisition equipment is shown in Figure 2, consisting of an SDG1022X Plus signal generator (SIGLENT, Shenzhen, China), SDS802X HD oscilloscope (SIGLENT, Shenzhen, China), and Aigtek ATA-214 high voltage amplifier (Aigtek, Xi’an, China). As the soil attenuation of ultrasonic waves is more obvious, high-frequency ultrasonic waves propagating in the soil are more likely to be affected by particle scattering, resulting in signal attenuation and distortion, so the ultrasonic transducer is selected as two low-frequency planar transducers with a center frequency of 50 kHz to enhance the ultrasonic penetration. Also, since transverse waves can only propagate in solids, and liquids and gases are usually still present in the soil, ultrasonic transducers capable of generating longitudinal waves are selected. The excitation signal of the signal generator is a 50 kHz pulse string with 12 pulses, a repetition period of 100 ms, and an amplitude of 4 Vpp. The amplitude of the excitation signal is amplified to 300 Vpp by a high-voltage amplifier to provide a sufficiently large voltage to stimulate the ultrasonic transducer to generate a strong enough ultrasonic pulse. Since the ultrasonic signal contains rich frequency components and rapidly changing waveform characteristics to make the signal waveform smoother and more accurate to avoid losing important information, the sampling rate of the oscilloscope is set to 1 Msa/s, which is much larger than the frequency of the ultrasonic signal and satisfies the Nyquist sampling theorem. The experiment used the ultrasonic transmission method to collect data from soil samples, and the ultrasonic transducer was fixed using a device with a guide rail to facilitate the contact and separation between the ultrasonic transducer and the soil samples. To ensure that the ultrasonic transducer can be completely affixed to the soil, we have coated both ultrasonic transducers with a coupling agent specially designed for ultrasonic flaw detection, and pushed the slider carrying the ultrasonic transducer to make the ultrasonic transducer and the soil gradually come into contact with each other, and when the oscilloscope waveforms are gradually visible and stable, the position of the slider will be fixed, and the data will be collected. There are 13 samples in this experiment. In order to have more data to train and test the soil porosity detection model, and in order to reduce the error caused by a single detection and improve the generalization of the model, each sample is collected every 30 s, a total of 10 times, and then exchange the position of the transmitter transducer and the receiver transducer and repeat the above operation. Finally, a total of 260 raw data points are collected in this experiment.

### 2.3. Ultrasonic Data Preprocessing

**Figure 3:**
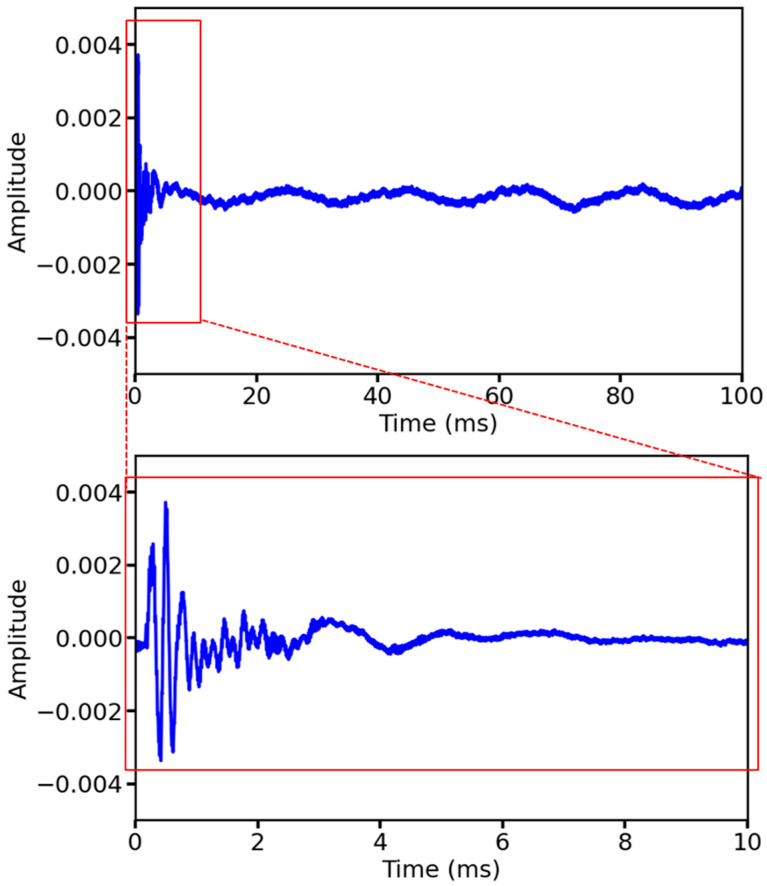
The segmentation process of the raw signal.

**Figure 4:**
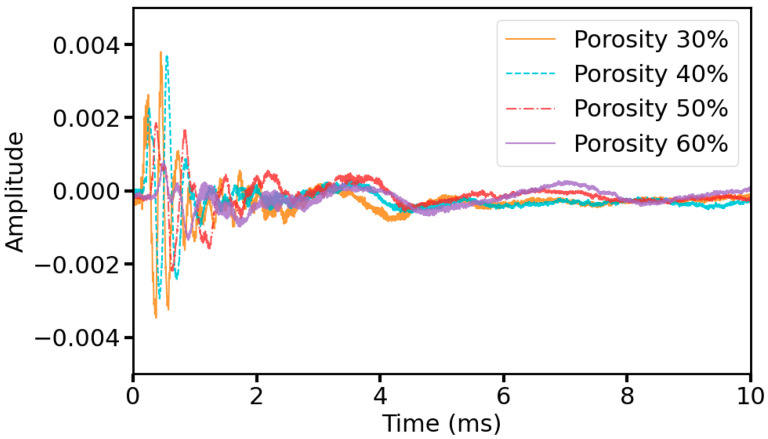
Original signal of some samples.

The collected ultrasonic data are 100 ms as a cycle because in ultrasonic nondestructive testing, the information related to the material properties can usually be analyzed from the first wave of the waveform and the subsequent attenuation until the smooth process [30], and to reduce the redundancy of the data, the complexity of the network model, and the memory pressure and accelerate the speed of the detection of the soil porosity, in this study, we selected the 0–10 ms portion of the waveform data for analysis and training, including 10,000 sampling points, which belong to long-term series data. Figure 3 shows the waveforms of the raw signal and segment signal (the segment signal is taken as the original signal in the subsequent description). From Figure 4, it is evident that with the increase in porosity, the amplitude of each peak of the waveform as a whole is reduced, and the appearance of the first wave is delayed, that is, the propagation speed of ultrasonic waves is slowed down, from which it can be illustrated that some features of ultrasonic waveforms and the soil porosity have a coupled relationship.

Deep learning models typically rely on large amounts of high-quality data to learn patterns and features. Still, in practice, the original dataset often has a limited size, a single distribution, or high labeling costs, which can easily lead to model overfitting. In this study, the amount of data collected is relatively small, and the collection environment is relatively singular, which cannot restore the complex environment of farmland, which may lead to the trained model not having qualified accuracy and anti-interference ability. Therefore, to increase the amount of data and improve model performance and generalization ability, this study introduces data augmentation techniques and expands the original signal dataset by adding Gaussian white noise, time shift transformation, and random perturbation. There are some subtle differences between random perturbations and white noise. Although they both belong to random noise, Gaussian white noise is a kind of random noise that conforms to a Gaussian (normal) distribution. This kind of noise is widely found in circuit systems. In contrast, random perturbation is an entirely random and irregular perturbation noise, which is used to simulate any unpredictable disturbances that may occur in the detection environment.

#### 2.3.1. Add Gaussian White Noise

**Figure 5:**
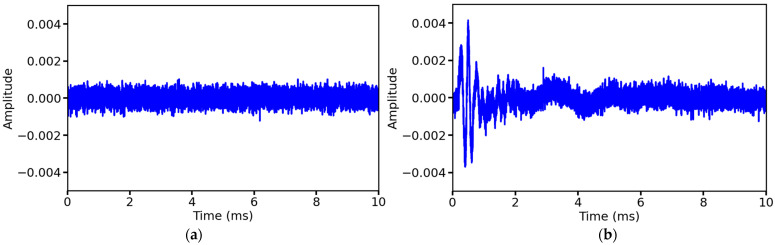
Gaussian white noise and the original signal with Gaussian white noise added: (**a**) Gaussian white noise with an SNR of 5; (**b**) Add Gaussian white noise with an SNR of 5 to the original signal.

Ultrasonic signals are susceptible to transducer electronic noise and environmental coupling noise, and the statistical properties of these noises are highly consistent with Gaussian white noise [31]. Gaussian white noise has the power spectral density characteristics of white noise in the frequency domain, i.e., the frequency components are uniformly distributed, the samples are independent of each other in the time domain, and its amplitude obeys a Gaussian distribution. By adding Gaussian white noise of controllable intensity to the original signal, the model can be forced to learn the physical nature of the noise-independent features while preserving the core acoustic features of the ultrasound detection signal. In this study, two types of Gaussian noise with positive and negative signal-to-noise ratios (SNRs) are added, with the noise intensity interval of 3 for positive SNRs set to 5, 8, 11, 14, 17, and 20, respectively, and the noise intensity interval of 2 for negative SNRs set to −4, −6, and −8, respectively, to avoid the noise being too large to be detached from the actual detection environment. The above noise settings cover the whole scene from the laboratory to the farmland, sufficient to simulate most noise disturbances in the real environment. A positive SNR indicates that the original signal strength is greater than the noise intensity, and the larger the absolute value, the smaller the relative noise intensity; a negative SNR indicates that the original signal strength is less than the noise intensity, and the larger the absolute value, the larger the relative noise intensity. Figure 5 shows the Gaussian white noise with an SNR of 5 and the signal after adding Gaussian white noise. Gaussian white noise is generated as follows:

1. Original signal power calculation:

(4) $$P_{\mathrm{signal}}=\frac{1}{N}\sum_{n=0}^{N-1}x{\left[n\right]}^{2},$$

where x[n] is the original signal, and N is the number of data sampling points, the average power is obtained by calculating the squared mean of the signal.

2. Noise power calculation:

(5) $$P_{\mathrm{noise}}=\frac{P_{\mathrm{signal}}}{10^{\frac{SNR}{10}}},$$

Based on a given signal-to-noise ratio (in decibels), the noise power is equal to the signal power divided by that linear value after converting it to a linear ratio.

3. Generate Gaussian white noise:

(6) $$w[n]∼N(0,\sqrt{P_{\mathrm{noise}}}),$$

Generate Gaussian white noise w[n] with mean 0, standard deviation $\sqrt{P_{\mathrm{noise}}}$, and variance $P_{\mathrm{noise}}$.

#### 2.3.2. Time Shift

**Figure 6:**
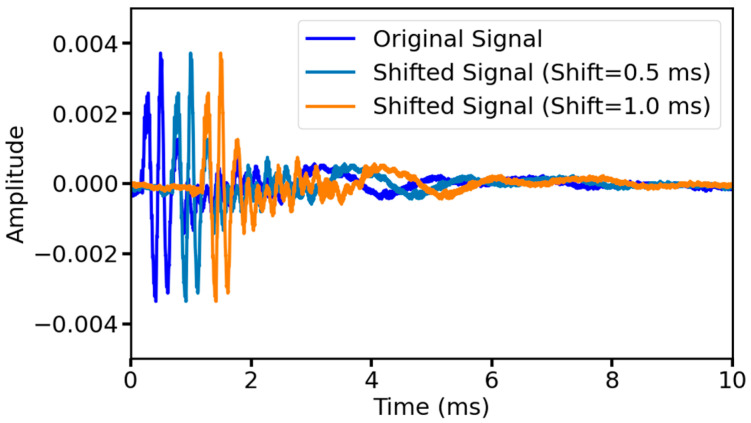
The signal before and after time shifting.

In actual measurements, ultrasonic signals may be offset in time due to equipment start-up time, signal transmission delays, etc. These temporal variations can be simulated by augmenting the original data with time-shifted data to make the model more robust. Moreover, even though the porosity of different soil samples may be the same, due to the different particle distributions of varying soil samples and the spatial heterogeneity of pore connectivity, the acoustic propagation paths may have differences in the bypass paths leading to local delays, and the time-shift operation can approximate the characterization of such natural fluctuations. To avoid the loss of information in the first wave by time-shifting the signal to the left, two degrees of time-shifting were applied to the right for all the original data in this study, namely, 0.5 ms to the right and 1 ms to the right, respectively. Figure 6 shows the original signal as well as the signal after time-shifting.

#### 2.3.3. Random Perturbation

**Figure 7:**
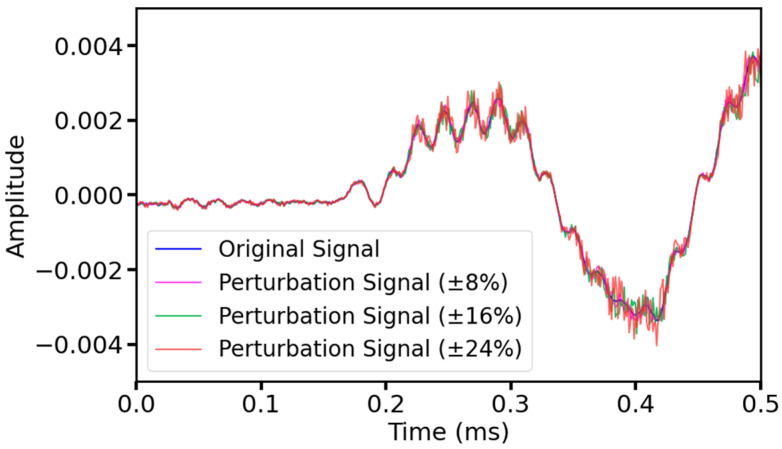
The signal before and after the amplitude perturbation.

Similarly, ultrasonic signals may be affected by various other unpredictable disturbances in real measurements, resulting in amplitude variations. By adding random amplitude perturbations, the model can be better adapted to these variations, improving the model’s performance in the face of disturbances and anomalies and enhancing the model’s robustness. Therefore, in this study, three scales of random perturbations were added to all the original data, namely 8%, 16%, and 24%, respectively, to allow the amplitude of each sample point in the ultrasound signal data to be randomly increased or decreased within the corresponding range, e.g., when the perturbation scale was 8%, the value of each sample point in the ultrasound sequence data was made to vary randomly within the range of ±8%. Figure 7 shows the original signal and the signal with the added amplitude perturbation.

After expanding the dataset using the three data augmentation techniques, there were 300 ultrasound signal data for each porosity, and a total of 3900 ultrasound signal data for 13 samples with different porosities were involved in the training, validation, and testing of the model. Among them are 260 original signals and 3640 augmented signals (2340 augmented signals with added white noise, 520 augmented signals with time-shift transformation, and 780 augmented signals with added random perturbation).

### 2.4. Introduction of Soil Porosity Testing Model

#### 2.4.1. Multi-Scale Feature Extraction

Since the ultrasonic detection signals of soils with different porosities have different frequency components and sequence features, if only a single-size convolution kernel is used, only a specific type of frequency feature can be extracted. It is not possible to evaluate the ultrasonic signals comprehensively. It may result in misjudgment by grouping signals with different porosities together, so to solve the problem, this study utilizes the multiscale feature extraction idea. Multi-scale CNN-LSTM, on the other hand, is a neural network model based on this idea, in which convolutional kernels of different sizes have unique significance in feature extraction [32]. Larger convolutional kernels have larger receptive field sizes, which can capture longer time-series patterns and global features such as long-term trends and overall shapes. These features are significant for identifying the macroscopic patterns and overall behavior of the ultrasound signal, which can reflect the overall structure and nature of the soil, and are suitable for extracting low-frequency and long-term dependent information while avoiding misjudgment due to local noise interference. In contrast, smaller convolution kernels have smaller receptive field sizes. They can capture local or short-term features in the ultrasound signal, such as short-term fluctuations, spikes, and local anomalies, which are essential for identifying the subtle changes and local defects in the signal, and are suitable for extracting high-frequency or short-term time series information, but there is considerable noise. Therefore, the use of different sizes of convolution kernels in the training of ultrasound signals can focus on the acoustic components of different frequencies, the high-frequency components reflect the microscopic porosity and the low-frequency components correspond to the macroscopic pore structure, and the multiscale model can process the acoustic responses of different frequencies in parallel, and then extract the multiscale time features, so it can adapt well to the complex non-uniform time series signals, and capture the characteristics of soil porosity related multilevel information, thus improving the robustness and accuracy of the model for porosity prediction. LSTM, as a specialized network for processing sequential data, can capture global and long-term dependent features. When combined with CNN’s ability to extract local features, it can further enhance the model’s feature expression ability, allowing it to capture temporal features of data more comprehensively [33]. Therefore, the combination of CNN and LSTM can synthesize spatial and temporal, global, and local information, and can significantly improve the flexibility and adaptability of the model when dealing with multidimensional time-series data, which can lead to excellent results in detecting soil porosity.

#### 2.4.2. MSRCNN-LSTMATT Based on Multi-Scale Feature Extraction

**Figure 8:**
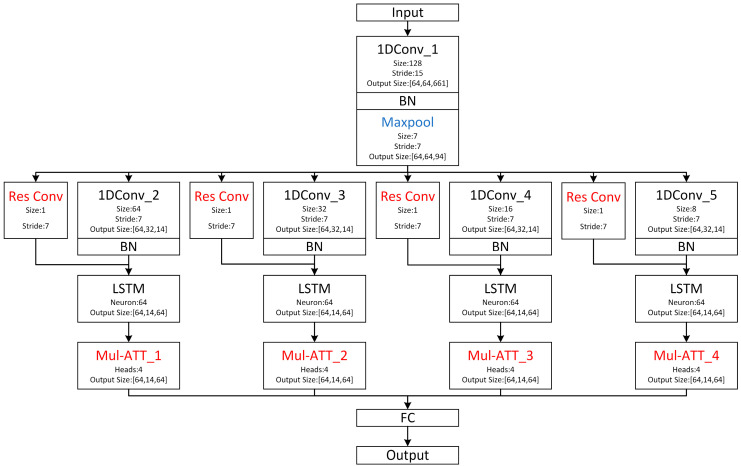
Structure of MSRCNN-LSTMATT.

Based on the multi-scale CNN-LSTM, we propose the MSRCNN-LSTMATT model, and the model structure is shown in Figure 8. The original ultrasound data contains a large number of sampling points; if the original data is directly trained, it will lead to too many model parameters, increasing the computational complexity, and at the same time, it may introduce a large amount of noise and redundant information, which leads to a decrease in the generalization ability of the model and thus overfitting. Using a large convolutional kernel to extract features from the data can capture a larger feature area, and better identify the global features in the data; at the same time, the data can be downscaled. However, the convolutional kernel is not as large as the better, too large convolutional kernel will lead to an increase in the amount of computation and memory consumption. Thus, the first 128 × 1 convolutional kernel should be used for feature extraction and downscaling of the original data, and at the same time, the step size will be set to 15 to further improve the dimensionality reduction capability of the convolutional kernel. The batch normalization layer is accessed behind the convolutional layer, which is responsible for normalizing the output of the convolutional layer, reducing the internal covariate bias, accelerating the training process, and at the same time, it can be viewed as a means of regularization to improve the model’s generalization, and finally accessing the maximal pooling layer, which downsamples the data while maintaining the salient features, thus reducing the computational complexity and the number of parameters. The input to the first convolutional layer is the original ultrasound signal, while the output is an abstraction of the original signal, i.e., the feature map of the original signal. After extracting the generalized global features, further extraction of more subtle and abstract high-level features is required. Thus, multi-scale feature extraction of the feature map using smaller convolutional kernels is needed. Multiple small convolution kernels of different sizes are required to extract features of different frequencies, so four small convolution kernels of different sizes are used to convolve the feature map to increase the diversity of features, and a batch normalization layer follows each small convolution kernel. It is worth noting that in a typical CNN, small convolutional kernels (e.g., 3, 5, 7) are sufficient to extract local features. However, in ultrasonic soil detection, an important phenomenon is the long-range dependence, which is manifested by the fact that a signal response that may be earlier in the soil waveform is associated with subsequent multipath propagation features. This temporal dependence may span tens or even hundreds of sampling points. While traditional small convolution kernels need to be stacked with multiple layers to expand the receptive field, large convolution kernels can replace the function of stacking multiple small convolution kernels to see a larger range of signal structure at once without stacking many layers, thus avoiding gradient vanishing and inefficiency, which makes it more powerful for remote coupling feature extraction between the first waveform and the subsequent scattered waveforms. Therefore, even the “small” convolutional kernel used in this study is only relative to the first convolutional kernel, which actually belongs to the category of large convolutional kernels. We chose convolutional kernel sizes of 8, 16, 32, and 64, which is the classical exponential growth combination, has the advantage of guaranteeing a doubling of the perceptual field at each layer, allows for a large perceptual field without too many convolutional kernels, and ensures that the network can perceive at all time scales. Although a large size of convolutional kernel helps to model long time-dependent features, the number of parameters and computational overhead will increase significantly, which may also bring overfitting risk when the data volume is limited; in this paper, the size of convolutional kernel is controlled to be around 100, which takes into account the remote dependence modeling and model generalization ability. The features extracted from each of these four convolutional kernels were then fed into four separate sets of LSTMs to further capture the dynamics and long-term dependencies of these features over time. Due to the high porosity of the soil, ultrasonic wave propagation in the soil will cause a large attenuation. The waveform is prone to distortion, so the model adds a multi-head self-attention mechanism after each group of LSTMs, which can directly compute the relationship between all positions in the sequence. Even if part of the region is distorted, the lost information can be inferred through the relationship between the front and back of the other regions to improve the degradation of the model’s performance caused by the distortion of the waveforms. The LSTM output sequence will be mapped into multiple different representation spaces. Each attention head focuses on capturing the information under different subspaces in the sequence [34], capturing key features in the ultrasound signals from multiple perspectives, enabling the model to comprehensively comprehend the complex patterns and structures in the sequence data, even in the presence of waveform distortion due to high porosity. After extracting features at different scales from different branches of the CNN-LSTM-ATT, they are spliced and fed into the fully connected layer so that the different features can complement each other. The final results of soil porosity detection are obtained. Due to the existence of deep network depth, to alleviate the problem of gradient vanishing [35] and make the network stable during the training process, a residual connection module is used in each set of CNNs, and a convolution kernel is added to the residual path for downsampling to ensure that the feature maps on the residual path are consistent with those on the main path in terms of spatial dimensions. Residual connections add inputs directly to outputs through jump connections, this structure allows the network to learn the residuals, i.e., the difference between the inputs and outputs, instead of trying to learn complex mappings directly from inputs to outputs, thus residual connections can more easily learn constant mappings, i.e., the outputs are the same as the inputs, and if some layers do not learn useful features, the network can at least pass the inputs directly through the residual connections, thus avoiding performance degradation due to increasing depth. The advantages of the MSRCNN-LSTMATT model are obvious: (1) The idea of multi-scale feature extraction enables the model to fully learn the features of the ultrasonic signals from different aspects, avoiding the one-sidedness of learning and thus evaluating the input signals more comprehensively and accurately. (2) Residual connection avoids the resulting degradation of model performance at deeper model depths, thus ensuring that the model’s ability to learn features in the ultrasound signal related to soil porosity is not degraded. (3) The multi-head self-attention mechanism can accurately extract useful information from the signal under the premise of serious distortion of ultrasonic signals in highly porous soils, avoiding the problem of model accuracy degradation due to signal attenuation and noise increase, and improving the model’s robustness. Some of the parameters of the model are shown in Table 2.

### 2.5. Performance Evaluation Metrics for the Model

In this study, the model’s performance is assessed using the coefficient of determination (R^2^) and the root mean square error (RMSE), which measure the model’s goodness-of-fit and the magnitude of error from different perspectives, respectively. The R^2^ measures the proportion of variance in the dependent variable that can be explained by the model, with a value between 0 and 1, which indicates the independent variable’s ability to explain the fluctuation of the dependent variable, and the closer its value is to 1, the better the explanatory ability of the model. RMSE measures the average difference between the predicted value and the actual value, reflecting the accuracy of the model’s prediction results; the smaller its value, the higher the model’s prediction accuracy. To more intuitively understand the proportion of the model error to the actual value, this study uses Percentage RMSE (referred to as RMSE in the rest of the paper). Its calculation formula is as follows:

(7) $$R^{2}=1-\frac{\sum_{i=1}^{n}{\left(y_{i}-{\hat{y}}_{i}\right)}^{2}}{\sum_{i=1}^{n}(y_{i}-\bar{y}{)}^{2}},$$

(8) $$RMSE=\sqrt{\frac{1}{n}\sum_{i=1}^{n}{\left(y_{i}-{\hat{y}}_{i}\right)}^{2}},$$

(9) $$Percentage\;RMSE=\frac{RMSE}{\bar{y}}\times 100\%,$$

where n is the number of soil samples, $y_{i}$ is the actual value of soil porosity, ${\hat{y}}_{i}$ is the predicted value of soil porosity, and $\bar{y}$ is the average value of soil porosity.

### 2.6. Visualization of Model Performance

In this study, we use T-SNE [36] to visualize model performance, a nonlinear dimensionality reduction algorithm commonly used for visualization of high-dimensional data, which helps to discover the clustering structure and anomalies in the data. The core idea is to use the high-dimensional probability distribution to calculate the data point similarity and embed it into the two-dimensional space through T-distribution, and finally optimize the low-dimensional embedding through KL scatter minimization, so that the data points can maintain the local structure of their high-dimensional space in the two-dimensional plane, thus visualizing the feature learning effect of the deep learning model. Specifically, T-SNE first computes the conditional probability distribution between data points in the high-dimensional space, i.e., for data points and, using a Gaussian distribution to define their similarity:

(10) $$p_{j|i}=\frac{\exp(-\frac{||x_{i}-x_{j}|{|}^{2}}{2\sigma_{i}^{2}})}{\sum_{k\neq i}\exp(-\frac{||x_{i}-x_{k}|{|}^{2}}{2\sigma_{i}^{2}})},$$

where $\sigma_{i}$ is an adaptive scaling parameter for point $x_{i}$ to ensure that the local structure of different density regions is reasonably well-reflected. Subsequently, the symmetric joint probability distribution is defined as:

(11) $$p_{\mathrm{ij}}=\frac{p_{j|i}+p_{i|j}}{2N},$$

where N is the total number of data points. In the low-dimensional space, T-SNE uses the T-distribution to define the similarity between data points $y_{i}$ and $y_{j}$. The long-tailed nature of the T-distribution can effectively avoid the “clustering” effect so that the low-dimensional embedding results can better maintain the overall structure of the data:

(12) $$q_{\mathrm{ij}}=\frac{{\left(1+||y_{i}-y_{j}|{|}^{2}\right)}^{-1}}{\sum_{k\neq l}{\left(1+||y_{k}-y_{l}|{|}^{2}\right)}^{-1}},$$

T-SNE optimizes the difference between high- and low-dimensional probability distributions by minimizing the Kullback–Leibler (KL) scattering with the following objective function:

(13) $$C=\sum_{i\neq j}p_{\mathrm{ij}}\log\frac{p_{\mathrm{ij}}}{q_{\mathrm{ij}}},$$

The gradient descent method is used to minimize the objective function C in order to optimize the embedding point $y_{i}$ and finally obtain a low-dimensional representation:

(14) $$\frac{\partial C}{\partial y_{i}}=4\sum_{j\neq i}(p_{\mathrm{ij}}-q_{\mathrm{ij}})(y_{i}-y_{j}){\left(1+||y_{i}-y_{j}|{|}^{2}\right)}^{-1},$$

## 3. Results and Discussion

**Figure 9:**
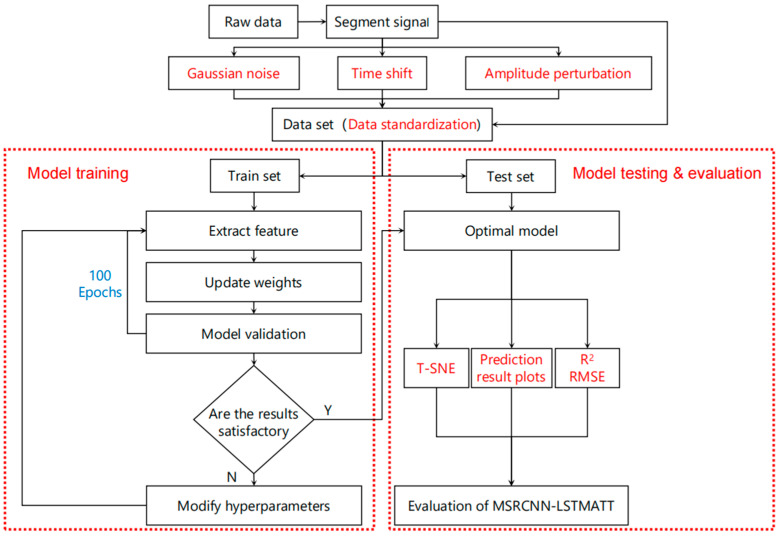
Flow chart of model training.

Before training the model, the data of each porosity label are randomly divided into the training set, validation set, and test set in the ratio of 8:1:1 to ensure that the model can fully and equally learn the features of ultrasound signal data of all porosities to obtain fairer experimental results. The model is implemented based on the PyTorch 1.13.0 framework, and the model training parameters are set as follows: the loss function is MSE because it is a widely used and valid objective for regression tasks. MSE penalizes larger errors more strongly and is particularly suitable for our normalized continuous porosity prediction, the optimizer is Adam because it has an adaptive learning rate mechanism and shows robust performance in a variety of tasks, especially in deep learning scenarios where the gradient is noisy or the dataset is small, the batch size is 64, which is a compromise between considering training stability and efficiency. A smaller batch size accelerates convergence and helps the model to get rid of local optima, but introduces more noise in the gradient estimation, while 64, a commonly used medium-sized batch size, strikes a good balance between computational efficiency and model generalization ability, the number of training iterations is 100 epochs, which is sufficient to ensure convergence while avoiding overfitting and wasting training time, according to the results of our multiple experiments, and the learning rate is an adaptive learning rate with an initial value of 0.0005 so that the model parameters can be adjusted more meticulously according to the gradient information during training. The model training flowchart is shown in Figure 9.

### 3.1. Ablation Experiment

**Figure 10:**
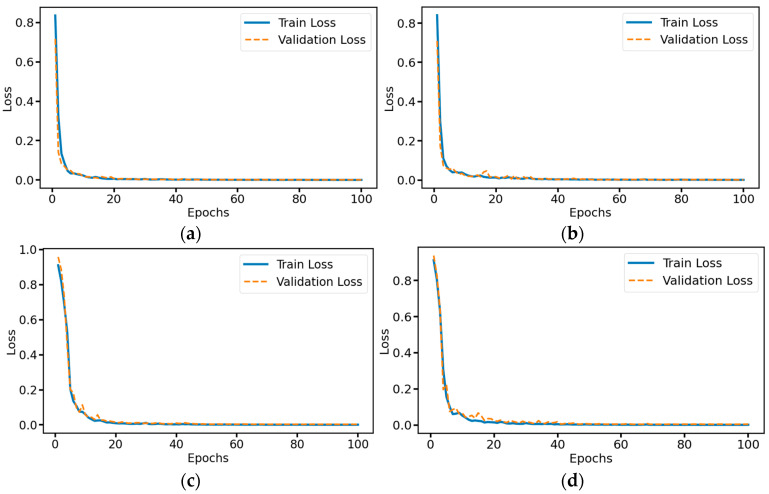
Training and validation loss curve of ablation experimental model: (**a**) Complete MSRCNN-LSTMATT; (**b**) MSRCNN-LSTMATT without residual connections; (**c**) MSRCNN-LSTMATT without multi-head self-attention; (**d**) MSRCNN-LSTMATT without residual connections and multi-head self-attention.

To verify the performance of our model and the optimization effect of different modules on the model, we conducted ablation experiments by removing the residual connection module, removing the multi-head self-attention mechanism module, and removing both the residual connection module and the multi-head self-attention mechanism module. As shown in Figure 10, the complete model’s training loss and validation loss decrease rapidly at the beginning of training and stabilize after about 20 epochs. The final loss value of the validation set stabilizes and stays near 0.001. This indicates that the model has a good fit on both the training and validation sets, and there is no obvious overfitting; after removing the residual connection module, although the training loss and validation loss of the model also decrease rapidly in the early stage of training, some fluctuation occurs after about 10 epochs, especially on the validation set loss. This indicates that after removing the residual connection, the training process of the model becomes less stable, and the epoch in which the final loss value reaches a smooth has also increased to some extent, leading to a decrease in the convergence speed; after removing the multi-head self-attention mechanism module, the model’s training loss and validation loss begin to decline slower at the beginning of the training process and fluctuations of a larger magnitude occur during the training process. This indicates that after removing the self-attention mechanism, the feature extraction ability of the model is more seriously affected, resulting in a slower and more unstable training process; after simultaneously removing the residual connection module and the multi-head self-attention mechanism module, the training loss and validation loss of the model decline most slowly at the beginning of the training process, and fluctuate the most violently during the whole training process, with the largest gap between the training loss and the validation loss. This indicates that the model’s training process becomes relatively unstable after removing both the residual connection and the multi-head self-attention mechanism, the convergence speed is severely slowed down, and the training effect is the worst overall.

**Figure 11:**
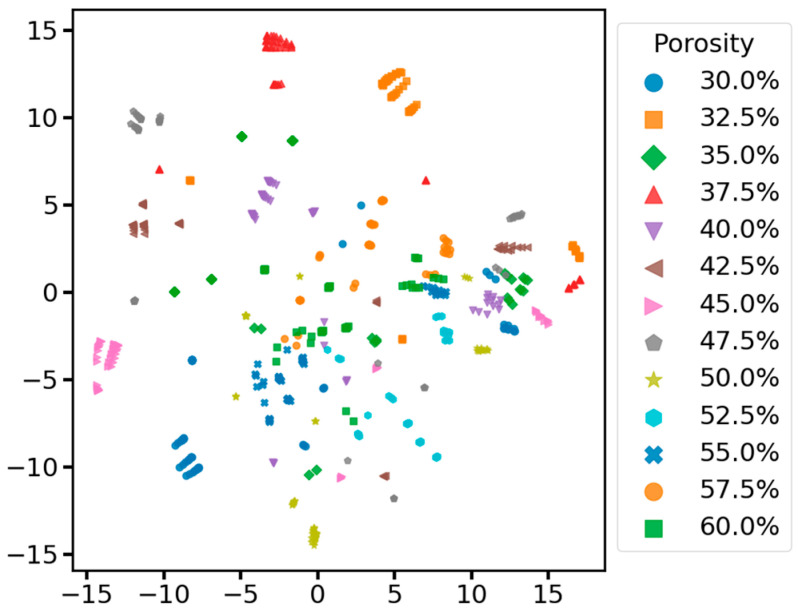
Visualization of input data in the test set based on T-SNE.

**Figure 12:**
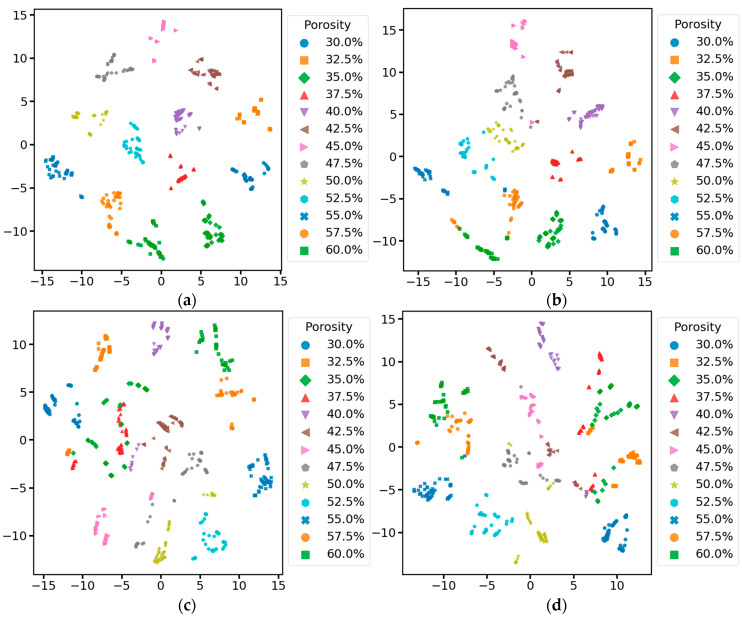
Visualization of data before the fully connected layer of different ablation models based on T-SNE: (**a**) Complete MSRCNN-LSTMATT; (**b**) MSRCNN-LSTMATT without residual connections; (**c**) MSRCNN-LSTMATT without multi-head self-attention; (**d**) MSRCNN-LSTMATT without residual connections and multi-head self-attention.

Meanwhile, to show the performance of our model more intuitively, we performed T-SNE visualization on the data before inputting the first convolutional layer and before inputting the fully connected layer in the test set of all models of the ablation experiment. To compare the distribution of the original data in the high-dimensional space and the distribution of the features extracted by the model from the original data in the high-dimensional space to judge the model’s performance. As shown in Figure 11, the distribution of original data with different porosities in the high-dimensional space is haphazard, and all categories of data are mixed without an obvious clustering structure, making it impossible to differentiate the different categories of data manually. After the feature extraction and learning of the data by each model in the ablation experiment, the data before inputting the full connectivity layer are all converged with the data of the same category to a certain extent in the high-dimensional space. According to the results in Figure 12, the samples with different porosities in the complete MSRCNN-LSTMATT model form a more obvious clustering structure in the figure. The separation between the clusters is very clear, which indicates that the model can effectively extract the features in the signals so that the samples of different categories are fully separated in the feature space. The samples of different porosities in the other three residual models still have some clustering structure in the figure, but the separation between different clusters is not as clear as in the complete model, and there is some overlapping mixing between samples of similar porosities. The model with the residual connection module removed shows a slight mixing of data in 57.5% and 60% porosities, the model with the multi-head self-attention mechanism module removed has a very serious mixing of data in 35% and 37.5% porosities, and the cluster structure and separation of the rest of the porosities are much worse than that of the complete model and the model with the residual connection module removed overall. The model with both the residual connection module and the multi-head self-attention mechanism module removed has the most significant drawback, with severe aliasing in most of the porosity data, and a relatively good cluster structure only in the 30%, 52.5%, and 55% porosity data. The results show that the residual connection module and the multi-head self-attention mechanism module play an essential role in the MSRCNN-LSTMATT model, which can significantly improve the feature extraction ability of the model and the classification performance of the data, in which the multi-head self-attention mechanism module plays a more important role because it is related to the ability of the model to capture and integrate different aspects of the contextual information at the same time, to enhance the modeling ability of the intrinsic structure of the complex data, and enabling the model to focus more comprehensively on the information about the soil porosity in different locations of the data so that it can differentiate between the data with different porosity.

**Figure 13:**
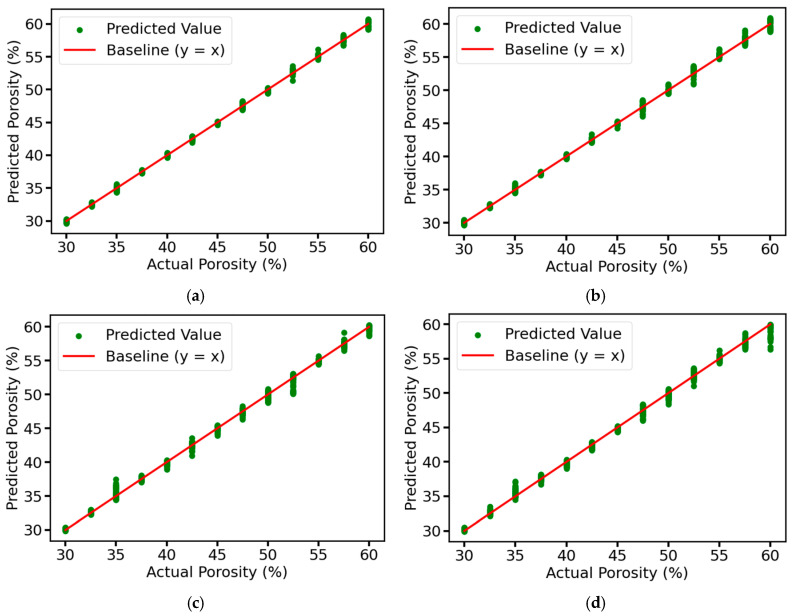
Prediction results of the test set for different ablation models: (**a**) Complete MSRCNN-LSTMATT; (**b**) MSRCNN-LSTMATT without residual connections; (**c**) MSRCNN-LSTMATT without multi-head self-attention; (**d**) MSRCNN-LSTMATT without residual connections and multi-head self-attention.

**Figure 14:**
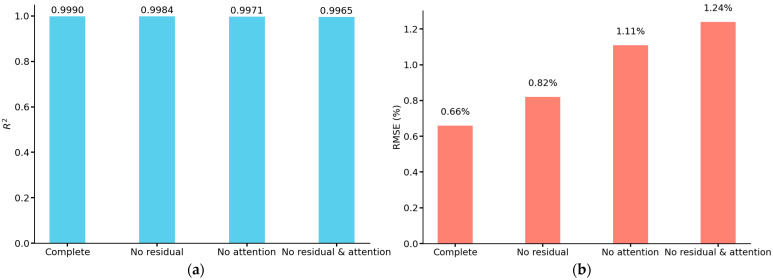
R^2^ and RMSE of the test set results for different ablation models: (**a**) R^2^ of different models; (**b**) RMSE of different models.

We use the prediction result plots to visualize the prediction results of each model for the test set in the ablation experiment, and use R^2^ and RMSE to evaluate the performance of the models. From Figure 13, it can be seen that the predicted values of the complete model are very close to the actual values, distributed in a narrow band, and most of the points are concentrated near the baseline, indicating that the model has very high prediction accuracy and fitting effect, and can accurately capture the intrinsic pattern of the data with almost no prediction error. As for the other three models, due to the removal of the corresponding modules, although the overall predicted values are still closer to the actual values, the distribution area is slightly wider than that of the complete model, resulting in some points deviating from the baseline by a longer distance, which may lead to the large deviation of the predicted values of some samples from the actual values. Figure 14 shows the values of R^2^ and RMSE of each model, the model with the residual connection module and multi-head self-attention mechanism module removed has an R^2^ of 0.9965, which is 0.0025 different from that of the complete model, both of which have good explanatory power. However, its RMSE is 1.24%, while the RMSE of the complete model is only 0.66%, which is reduced by about 46.77%, and the accuracy of the prediction results is higher. According to the results in Figure 14, it can be seen that the error is increased by about 24.24% after removing the residual connection module. In comparison, the error is increased by about 68.18% after removing the multi-head self-attention mechanism module. Although both are indispensable, the multi-head self-attention mechanism module plays a more critical role in the model than the residual connection module, which can more significantly improve the model’s feature extraction ability and prediction performance.

**Figure 15:**
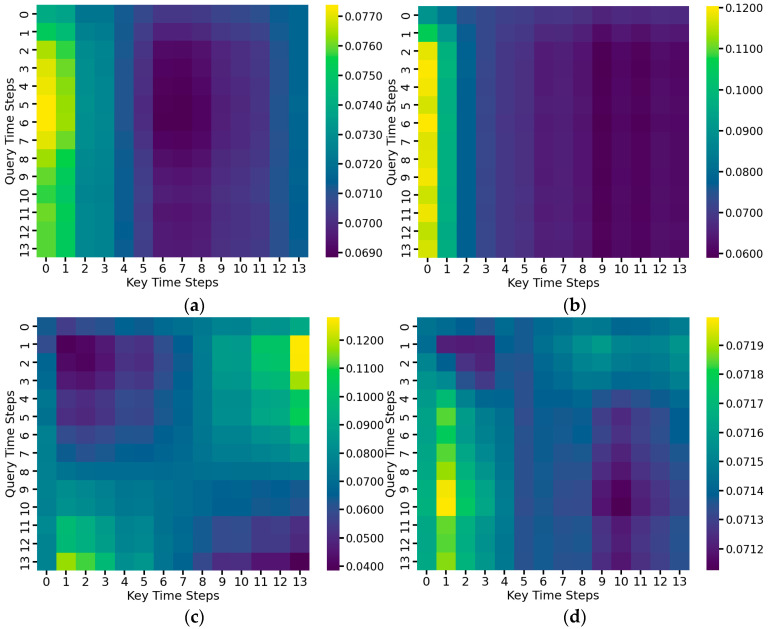
Heatmap of average multi-head attention weights for each attention layer of the MSRCNN-LSTMATT model: (**a**) Average weight of the first attention mechanism layer; (**b**) Average weight of the second attention mechanism layer; (**c**) Average weight of the third attention mechanism layer; (**d**) Average weight of the fourth attention mechanism layer.

To further analyze the model’s attention mechanism on temporal signals, this paper visualizes each attentional layer’s average multi-head attentional weights of the complete MSRCNN-LSTMATT model as shown in Figure 15. In the self-attention mechanism, the data of each time step will act as both Query, the viewpoint from which the current time step wants to get information, and Key, the information provided by all other time steps. The attention mechanism is to calculate how much attention each Query has paid to the Key at all time points. The horizontal axis in the heatmap represents the temporal position (Key) in the input sequence, the vertical axis represents the position (Query) where the prediction is currently being made, and the color shades indicate the intensity of the model’s attention on each time step during the prediction process.

As can be seen from the figure, the first and second attention mechanism layers mainly focus on the beginning of the signal sequence, which indicates that the model considers these time periods to be the most informative for porosity prediction. In contrast, in the third and fourth attention mechanism layers, the distribution of attention is more dispersed, suggesting that the deeper model is beginning to integrate a broader range of global information. This hierarchical attention mechanism shows the model’s ability to adapt itself in the time dimension, and also helps to improve the model’s interpretability and generalization.

**Figure 16:**
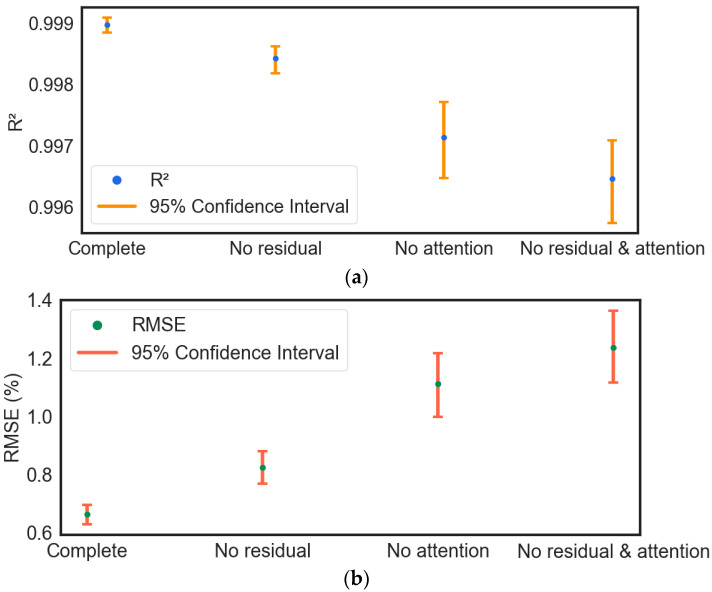
Visualization of 95% confidence intervals for R^2^ and RMSE in ablation experiments: (**a**) 95% confidence interval for R^2^; (**b**) 95% confidence interval for RMSE.

Meanwhile, we utilize bootstrapping to report the 95% confidence intervals of R^2^ and RMSE for the ablation experiments, as shown in Table 3 with Figure 16, from which it can be seen that the confidence intervals for the complete model are smaller. At the same time, those for the removal of residual linkage and the multi-head self-attention mechanism are larger. This means the complete MSRCNN-LSTMATT model has less fluctuation, more stable model performance, and higher confidence in the results.

### 3.2. Model Comparison

To further demonstrate the superior performance of our model, we compare it with other advanced models, including WDCNN [32], Hierarchical LSTM [37], and 1D-CNN [38], which also process vibration signals, and a popular deep neural network, 1D-Resnet18 [35]. Among them, WDCNN is the first deep convolutional neural network model with wide convolutional kernels, Hierarchical LSTM is a neural network model with three LSTM layers, 1D-CNN is a convolutional neural network model with three convolutional layers and two average pooling layers, and 1D-Resnet18 is a network model that changes the convolutional layer to a one-dimensional convolution based on the original Resnet18 model. The structure of these models is shown in Table 4, where Conv64 denotes a convolutional layer with a convolutional kernel size of 64, Maxpool2 denotes a maximum pooling layer with a pooling window size of 2, AveragePool8 denotes an average pooling layer with a pooling window size of 8, FC100 denotes a fully connected layer with a neuron number of 100, LSTM64 denotes an LSTM layer with 64 neurons, and the same for the rest of the layers.

**Figure 17:**
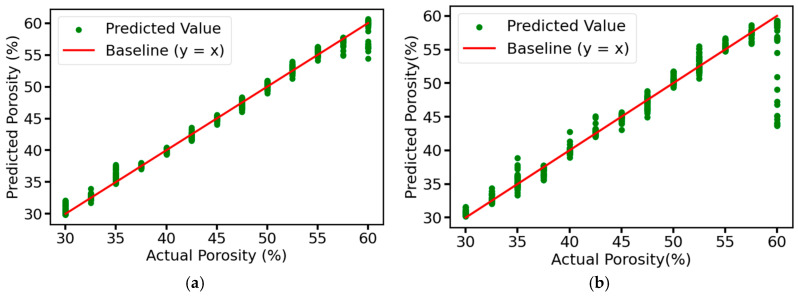

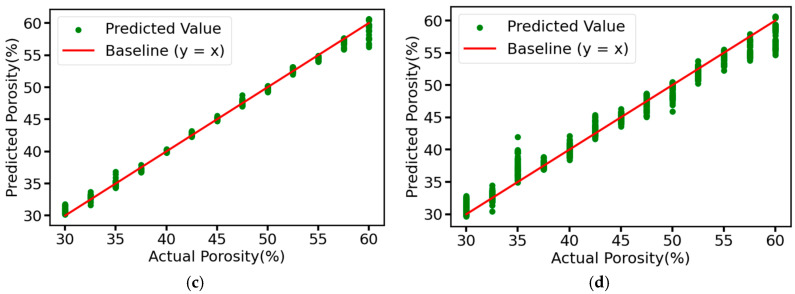
Prediction results of the test set for contrast models: (**a**) WDCNN; (**b**) Hierarchical LSTM; (**c**) 1D-CNN; (**d**) 1D-Resnet18.

**Figure 18:**
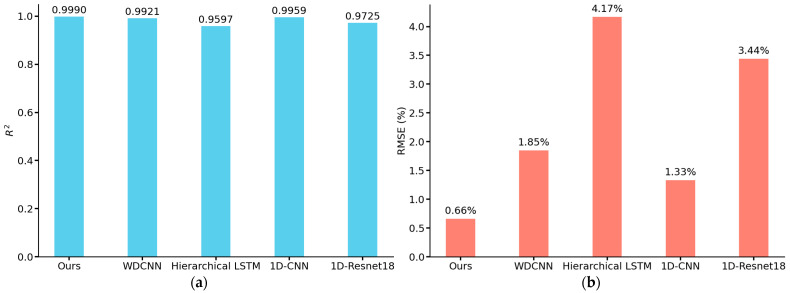
R^2^ and RMSE of the test set results for contrast models: (**a**) R^2^ of our model and contrast models; (**b**) RMSE of our model and contrast models.

Figure 17 shows the prediction results of the comparison model, and Figure 18 shows the R^2^ and RMSE values of our model and the comparison model. It is noteworthy that the comparison models all have satisfactory results in the task of detecting soil porosity. Among them, the prediction results of WDCNN and 1D-CNN are more accurate relative to Hierarchical LSTM and 1D-Resnet18, and the distribution of data points is closer to the neighborhood of the baseline. This is because Hierarchical LSTM, although using LSTM layers commonly used for sequence data, lacks a convolutional layer, thus limiting its feature extraction capability; whereas Resnet18, as a generalized image convolutional neural network, may not be efficient enough in processing 1D signals in its 1D-convolutional version, and its architecture is not able to adequately capture the time-series features in the data. MSRCNN-LSTMATT makes its prediction results better than the four compared models due to the full combination of the multi-scale hierarchical feature extraction capability of multiple convolutional layers and the ability of the LSTM layer to deal with time series dependencies in the data. Figure 4 also demonstrates that the propagation of ultrasonic signals in highly porous soils can lead to phenomena such as reduced wave amplitude and increased attenuation, which leads to significant distortion of ultrasonic waveforms, which become blurred or difficult to recognize, and thus may lead to noise components masking useful signal features, further increasing the difficulty of feature extraction. The multi-head self-attention mechanism module in MSRCNN-LSTMATT can consider the information of all positions in the sequence at the same time, which is very helpful in dealing with the waveform variation and feature blurring caused by high porosity, and can focus on the important parts of the signal even in the case of waveform deterioration caused by high porosity, thus suppressing the influence of the noise and extracting the functional features, which solves the problem that the other models have no effect on the high porosity soils with generally low detection accuracy.

## 4. Conclusions

This study proposes an improved multi-scale CNN-LSTM soil porosity detection model based on large convolution kernels for soil ultrasonic detection data with many sampling points and serious distortion. The model mainly performs feature extraction on the collected ultra-long time series data by utilizing multiple large convolution kernels of different sizes to obtain the features of different scales in the data and improve the accuracy of soil porosity test results. The model achieves excellent results in the self-constructed soil dataset, and the residual connection module and the multi-head self-attention mechanism module in the model can further improve the detection performance of the multi-scale CNN-LSTM network as the backbone, with the R^2^ reaching 0.9990 and the RMSE decreasing by 46.77% to only 0.66%. Meanwhile, the improvement is huge compared with other advanced detection models, so this soil porosity detection method has the advantages of high accuracy, strong anti-interference, low cost, and has a broad application prospect.

It should be noted, however, that the datasets used in this study were taken from soil samples constructed under controlled conditions with a limited range of texture types and water contents that do not yet cover more complex or diverse field soil conditions. Although the model showed better prediction performance on this self-constructed dataset than the other models, its generalization ability to other texture types, different water content statuses, or real field soils still needs further verification. Future work will consider introducing more types and sources of soil samples to enhance the adaptability and usefulness of the model.

## Figures and Tables

**Table 1:** Ultrasonic testing tasks based on conventional machine learning.

Ultrasonic Testing Task	Input Data	Model	Model Performance	
Furnace tube carburization damage detection [14]	The features grouped in a 40 × 22 matrix are extracted using a set of 22 FFT coefficients	Gaussian Naive Bayes	Accuracy: 99.2%	
		Kernel Naive Bayes	Accuracy: 97.5%	
		Subspace Discriminant	Accuracy: 90.8%	
Quantitative evaluation of corrosion deterioration of rubberized concrete steel [15]	Ultrasonic amplitude, ultrasonic velocity, and rubber content	Bayesian ridge regression	R^2^: 0.867	
			MAE (g): 2.893	
			MSE (g^2^): 12.735	
		K-nearest neighbor	R^2^: 0.970	
			MAE (g): 1.256	
			MSE (g^2^): 2.880	
		Random forest	R^2^: 0.971	
			MAE (g): 1.221	
			MSE (g^2^): 2.820	
		Voting	R^2^: 0.974	
			MAE (g): 1.176	
			MSE (g^2^): 2.431	
		Bagging	R^2^: 0.972	
			MAE (g): 1.221	
			MSE (g^2^): 2.706	
		Stacking	R^2^: 0.973	
			MAE (g): 1.220	
			MSE (g^2^): 2.781	
Classification of defects from ultrasonic scanning of A380 aircraft components [16]	C-scan images storing Region of Interest labels (rectangle-position, pixel area) and scene labels (defective and good)	HoG-Linear SVM	Accuracy: 99.0%	
			Recall: 0.9919	
			Precision: 0.9880	
			F1-score: 0.984	
			ROC-AUC: 1.00	
		SURF-Decision Fine Tree	Accuracy: 97.9%	
			Recall: 0.9839	
			Precision: 0.9700	
			F1-score: 0.970	
			ROC-AUC: 0.92	
Predicting the content of sand, silt, and clay fractions in soils [19]	Ten characteristic points representative of the curve extracted from the signals obtained from the regression experiments on all soils, and the particle ratios corresponding to the regression experiments on different particles	Support Vector Regression	Sand	R^2^: 0.52
				MAE: 12.42
				MSE: 299.68
			Silt	R^2^: 0.10
				MAE: 13.51
				MSE: 310.29
			Clay	R^2^: 0.38
				MAE: 11.69
				MSE: 221.22
Developed a non-destructive method of evaluating soil moisture using a contactless ultrasonic system [20]	Normalized amplitude, energy of Rayleigh leakage waves	Random forest	R^2^ ≥ 0.98	
			RMSE ≤ 0.0089	

**Table 2:** Partial model parameters of MSRCNN-LSTMATT.

Layer Name	Kernel/Window Size	Stride	Padding	Output Channels	Neuron	Heads	Activation Function
1DConv_1	128	15	NO	64	/	/	ReLU
Maxpool	7	7	NO	64	/	/	/
1DConv_2	64	7	YES	32	/	/	ReLU
1DConv_3	32	7	YES	32	/	/	ReLU
1DConv_4	16	7	YES	32	/	/	ReLU
1DConv_5	8	7	YES	32	/	/	ReLU
Res Conv	1	7	NO	32	/	/	/
LSTM	/	/	/	/	64	/	ReLU
Mul-ATT(ALL)	/	/	/	/	/	4	/

**Table 3:** 95% confidence intervals(CI) for R^2^ and RMSE in ablation experiments.

Model	R^2^	RMSE
Complete	0.9990, CI: (0.9989–0.9991)	0.66%, CI: (0.63–0.70%)
No residual	0.9984, CI: (0.9982–0.9986)	0.82%, CI: (0.77–0.88%)
No attention	0.9971, CI: (0.9965–0.9977)	1.11%, CI: (1.00–1.22%)
No residual & attention	0.9965, CI: (0.9957–0.9971)	1.24%, CI: (1.12–1.36%)

**Table 4:** Structure of contrast models.

Contrast Models	Structure
WDCNN	Conv64--Maxpool2--Conv3--Maxpool2--Conv3--Maxpool2--Conv3--Maxpool2--Conv3--Maxpool2--FC100--FC1
Hierarchical LSTM	LSTM64--Dropout--LSTM32--Dropout--LSTM32--Dropout--FC1
1D-CNN	Conv64--AveragePool8--Conv128--Conv128--AveragePool2--Dropout--FC1024--Dropout--FC1
1D-Resnet18	The model is too large to be displayed, please refer to paper [35]

## Data Availability

The data presented in this study are available on request from the corresponding author.
